# Percutaneous Endoscopic Necrosectomy of Walled-Off Pancreatic and Peripancreatic Necrosis

**DOI:** 10.3390/jcm15020470

**Published:** 2026-01-07

**Authors:** Mateusz Jagielski, Agata Chwarścianek, Damian Dudek, Jacek Piątkowski, Marek Jackowski

**Affiliations:** 1 Department of General, Gastroenterological and Oncological Surgery, Ludwik Rydygier Regional Hospital, 87-100 Toruń, Poland; matjagiel@gmail.com (M.J.); jacek.piatkowski@cm.umk.pl (J.P.);; 2 Department of Perioperative Dentistry, Faculty of Medicine, Ludwik Rydygier Collegium Medicum in Bydgoszcz, Nicolaus Copernicus University, 87-100 Toruń, Poland

**Keywords:** pancreatic necrosis, percutaneous access, endoscopic treatment

## Abstract

**Background**: Minimally invasive approaches for managing complications of acute necrotizing pancreatitis have advanced significantly in recent decades. When extensive walled-off pancreatic or peripancreatic necrosis is present, a single transluminal access may be insufficient. This study aimed to prospectively evaluate the effectiveness and safety of a novel percutaneous endoscopic necrosectomy technique used as an adjunct to transmural drainage in patients with symptomatic walled-off necrosis. **Methods**: A total of 513 consecutive patients with symptomatic walled-off pancreatic or peripancreatic necrosis treated between 2018 and 2025 at a single tertiary center in Poland were included. All patients underwent minimally invasive endoscopic management. Among them, a subgroup required additional percutaneous drainage. The innovative technique involved creating retroperitoneal percutaneous access to the necrotic cavity, enlarging the tract, and placing a self-expanding metal stent to allow passage of the endoscope for percutaneous endoscopic necrosectomy. **Results**: Additional percutaneous drainage was necessary in 39/513 patients (7.6%). Of these, 9/39 (23.1%) patients (2 women, 7 men; mean age 46.7 years) underwent percuaneous endoscopic necrosectomy. The mean size of the necrotic collection was 25.96 cm. Active percutaneous drainage during ongoing transmural endotherapy lasted a median of 15 days. Patients underwent an average of 3.12 necrosectomy sessions. Treatment-related complications occurred in 2/9 patients (22.22%). Clinical and long-term success were each achieved in 8/9 patients (88.89%). **Conclusions**: Percutaneous endoscopic necrosectomy is a promising minimally invasive therapeutic option for extensive walled-off pancreatic and peripancreatic necrosis, particularly when necrosis extends into the pelvic region. However, clinical evidence remains limited and further studies are needed.

## 1. Introduction

The treatment of complications of severe acute pancreatitis continues to be a major clinical problem and a challenge to even the most experienced pancreatologists. Severe acute pancreatitis is associated with local complications in the form of pancreatic and peripancreatic fluid collections and is characterized by extremely variable and unpredictable dynamics, high risk of complications, and high mortality rates [1,2,3]. Thanks to the development of minimally invasive techniques for use in the treatment of acute necrotizing pancreatitis complications, improvement in the treatment outcomes of patients with pancreatic necrosis has been observed in recent years [4,5,6,7]. Minimally invasive methods for the treatment of pancreatic and peripancreatic necrosis include procedures performed using an endoscope, laparoscope, or nephroscope, which facilitate transperitoneal, retroperitoneal, transmural, or transpapillary access to the necrosis.

The transmural endoscopic access is the preferred method for accessing necrotic collections if the distance between the wall of the collection and the wall of the gastrointestinal tract does not exceed 40 mm [8,9]. In other cases, i.e., if the distance between the necrotic collection and the gastrointestinal tract is greater than 40 mm, percutaneous access remains the treatment of choice, with extraperitoneal approach providing the preferred access for necrosectomy procedures, including the following:

- Sinus tract endoscopy (referred to in the literature as minimally invasive retroperitoneal pancreatic necrosectomy (MIRPN) or minimal access retroperitoneal pancreatic necrosectomy (MARPN));
- Video-assisted retroperitoneal debridement (VARD) [10,11].

In the aforementioned techniques, extraperitoneal access to the necrotic cavity is achieved by prior placement of a percutaneous drain under radiographic guidance. The sinus tract endoscopy technique involves gradual widening of the canal formed at the site following the insertion of the percutaneous drain until it reaches a diameter of 30 Fr; this is followed by a rigid nephroscope or flexible endoscope being inserted into the necrotic cavity and the necrotic contents being flushed and aspirated [12,13]. The sinus tract endoscopy technique can be used as an adjunctive treatment following a prior open necrosectomy. Video-assisted retroperitoneal debridement, a hybrid between sinus tract endoscopy and open necrosectomy from extraperitoneal access, is another technique for minimally invasive treatment of pancreatic necrosis. The VARD technique involves an incision being made near the percutaneous drain, the end of which is placed within the lumen of the necrotic collection, followed by the fluid part being removed using a suction system and the necrotic tissue being removed using long forceps. The procedure is performed under the guidance of a camera inserted into the necrotic cavity through a laparoscopic access port [11,14,15].

The currently acknowledged step-up strategy for the treatment of acute pancreatitis complications consists of the necrotic areas being gradually expanded by transmural approach or percutaneous surgical techniques [16,17].

With the growing role of endoscopic therapy, percutaneous endoscopic necrosectomy (PEN) has emerged as an innovative technique that bridges the advantages of percutaneous access with direct endoscopic visualization of the necrotic cavity. PEN is particularly valuable when transmural drainage is ineffective or anatomically impossible—especially when the distance between the necrotic collection and the gastrointestinal wall exceeds 40 mm or when collections are positioned laterally or posteriorly, limiting safe access via the stomach or duodenum. The method allows repeated debridement sessions using standard endoscopic tools and offers better visualization and selective removal of necrotic tissue compared with blind percutaneous approaches. Early reports suggest that PEN may reduce the need for open surgery, facilitate faster clearance of necrosis, and shorten drainage duration. Moreover, it may be combined with other modalities used in the step-up pathway, positioning PEN as a complementary technique within the spectrum of minimally invasive interventions.

However, despite its promising potential, PEN has not yet gained widespread application. The number of publications remains limited, mostly involving small patient groups and heterogeneous protocols, which makes it difficult to develop standardized recommendations. Uncertainties persist regarding optimal timing, technical aspects, number of sessions required, and long-term outcomes compared with other minimally invasive methods. This creates a distinct evidence gap and emphasizes the need for further well-designed clinical research assessing PEN as an alternative or adjunct to established techniques [18].

Therefore, the aim of this study was to prospectively evaluate the efficacy and safety of percutaneous endoscopic necrosectomy in patients with symptomatic walled-off pancreatic necrosis.

## 2. Materials and Methods

The study group consisted of patients with symptomatic walled-off pancreatic necrosis who had undergone PEN in the course of endoscopic percutaneous endoscopic drainage at the Department of General, Gastroenterological, and Oncological Surgery at the L. Rydygier Regional Hospital in Toruń. A total of 513 consecutive patients with symptomatic walled-off pancreatic or peripancreatic necrosis treated between 2018 and 2025 at a single tertiary center in Poland were included. All patients underwent minimally invasive endoscopic management. Among them, 39 required additional percutaneous drainage. The innovative technique, involving creating retroperitoneal percutaneous access to the necrotic cavity, enlarging the tract, and placing a self-expanding metal stent to allow passage of the endoscope for percutaneous endoscopic necrosectomy, was performed in 9 patients.

All patients were initially subjected to percutaneous drainage under ultrasound guidance using the 16 Fr, 41 cm Thal-Quick Abscess Drainage Set (COOK Medical) so as to establish multiple access points to the necrotic cavity. In cases of no clinical improvement, defined as the collection size regressing to less than 50% of baseline being observed after one week of percutaneous drainage, the patients qualified for the PEN procedure.

**Inclusion criteria:**

− Diagnosed walled-off pancreatic necrosis (WOPN) confirmed by contrast-enhanced CT.
− Symptomatic necrotic collection presenting with persistent abdominal pain, fever, SIRS, infection confirmed radiologically or microbiologically, gastrointestinal/biliary obstruction, or organ dysfunction associated with the necrotic collection.
− Lack of clinical response to the previous treatment.
− Collection not amenable to transmural endoscopic access (distance from gastrointestinal wall > 40 mm or unfavorable anatomical localization).
− Age ≥ 18 years.

**Exclusion criteria:**

− Acute necrotic collection < 4 weeks from onset without maturation of the collection wall.
− Hemodynamic instability or severe organ failure contraindicating minimally invasive intervention.
− Inability to establish a safe percutaneous access route.
− Uncorrected coagulopathy (e.g., INR > 1.5, platelet count < 50,000/µL).
− Conditions requiring immediate open surgery (e.g., suspected bowel perforation, abdominal compartment syndrome, uncontrolled hemorrhage).

**Figure 1:**
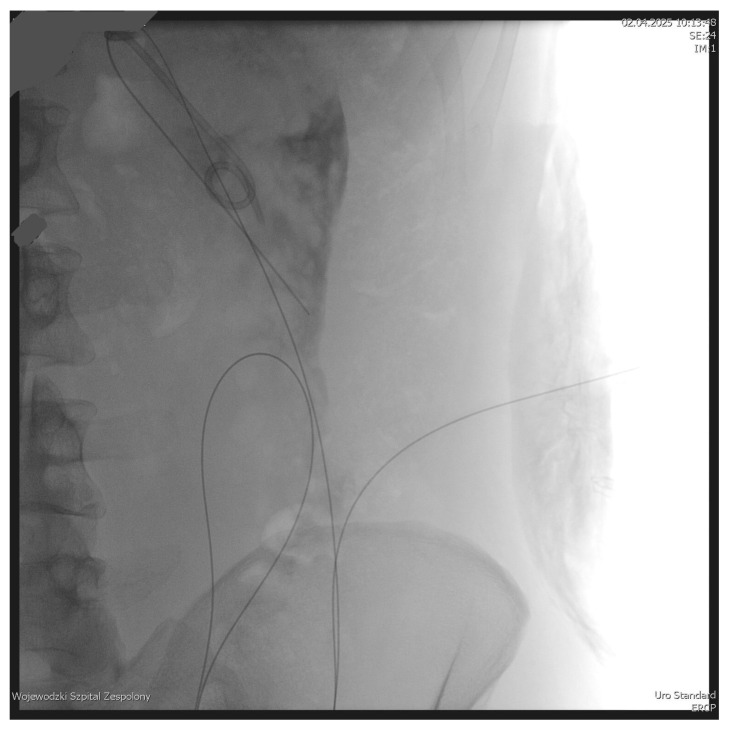
Looped guidewire within the lumen of the WOPN cavity. Source: Department of General, Gastroenterological, and Oncological Surgery, L. Rydygier Regional Hospital in Toruń.

**Figure 2:**
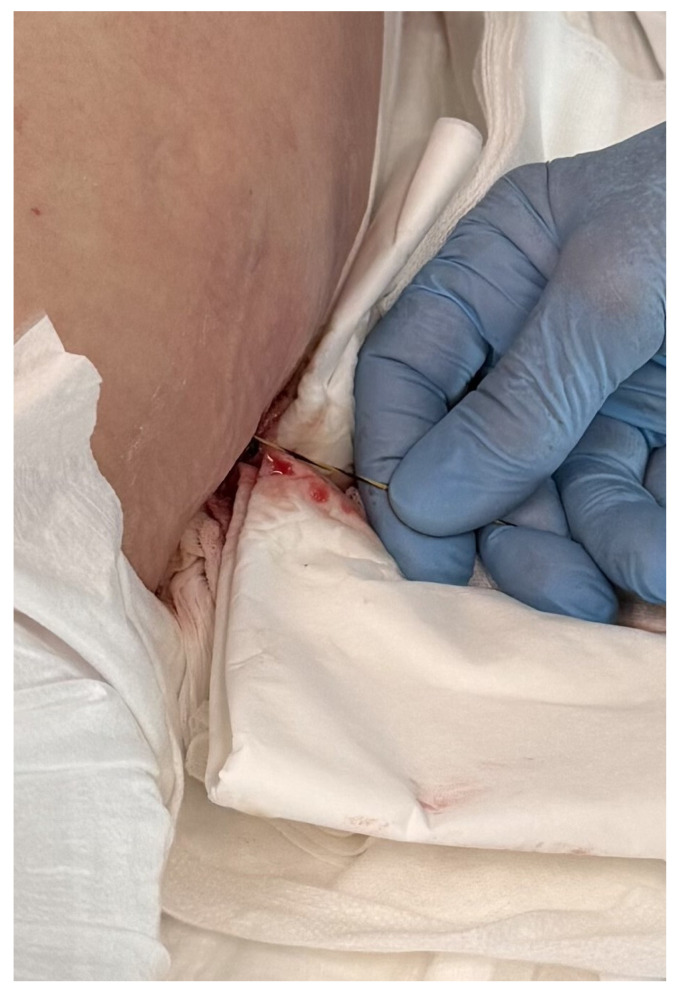
Guidewire percutaneously inserted into the lumen of the WOPN collection. Source: Department of General, Gastroenterological, and Oncological Surgery, L. Rydygier Regional Hospital in Toruń.

**Figure 3:**
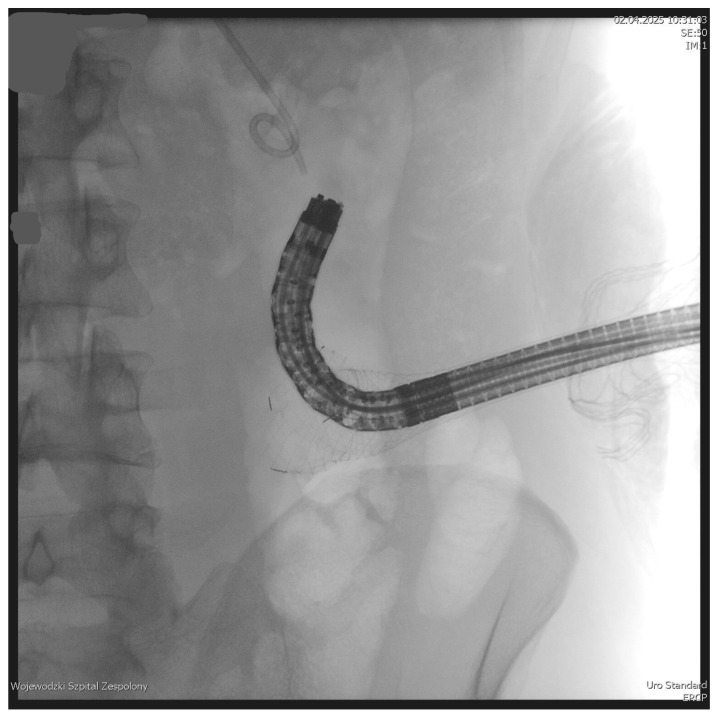
Endoscope within the lumen of the WOPN cavity. Source: Department of General, Gastroenterological, and Oncological Surgery, L. Rydygier Regional Hospital in Toruń.

**Figure 4:**
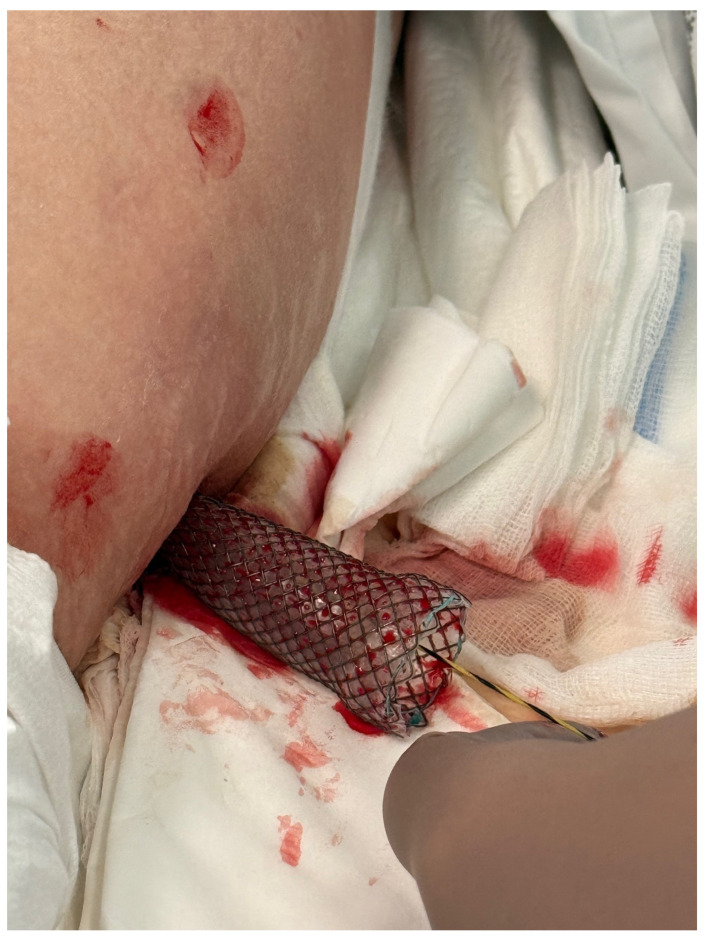
A fully coated, self-expandable esophageal stent inserted percutaneously. Source: Department of General, Gastroenterological, and Oncological Surgery, L. Rydygier Regional Hospital in Toruń.

**Figure 5:**
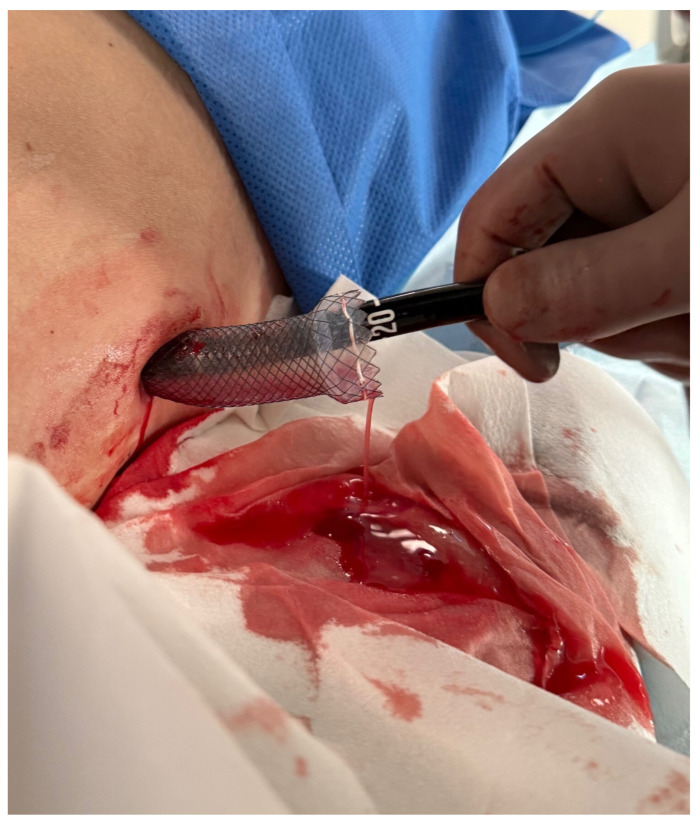
Percutaneous endoscopic necrosectomy procedure. Source: Department of General, Gastroenterological, and Oncological Surgery, L. Rydygier Regional Hospital in Toruń.

**Figure 6:**
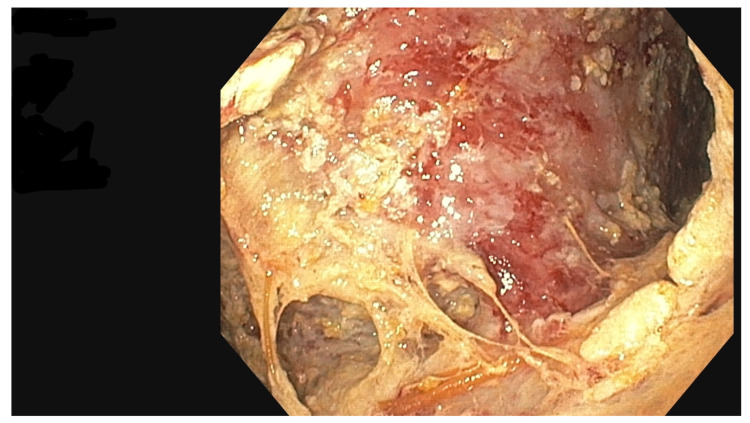
Walled-off pancreatic necrosis collection. Source: Department of General, Gastroenterological, and Oncological Surgery, L. Rydygier Regional Hospital in Toruń.

**Figure 7:**
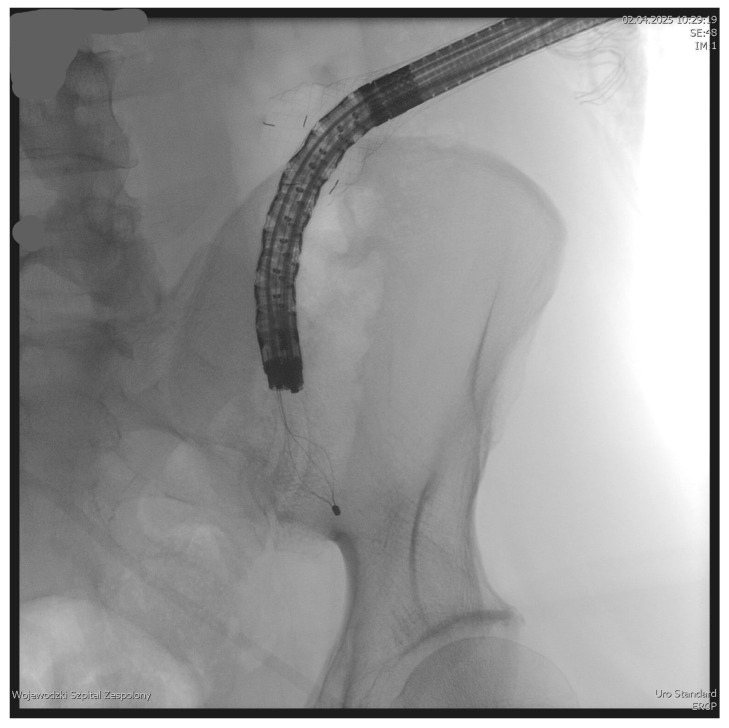
Percutaneous endoscopic necrosectomy using a Dormia basket. Source: Department of General, Gastroenterological, and Oncological Surgery, L. Rydygier Regional Hospital in Toruń.

**Figure 8:**
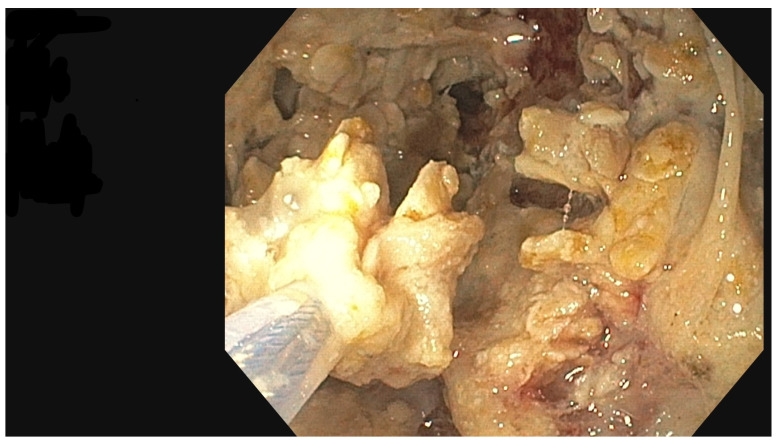
Percutaneous endoscopic necrosectomy procedure, endoscopic view. Source: Department of General, Gastroenterological, and Oncological Surgery, L. Rydygier Regional Hospital in Toruń.

**Figure 9:**
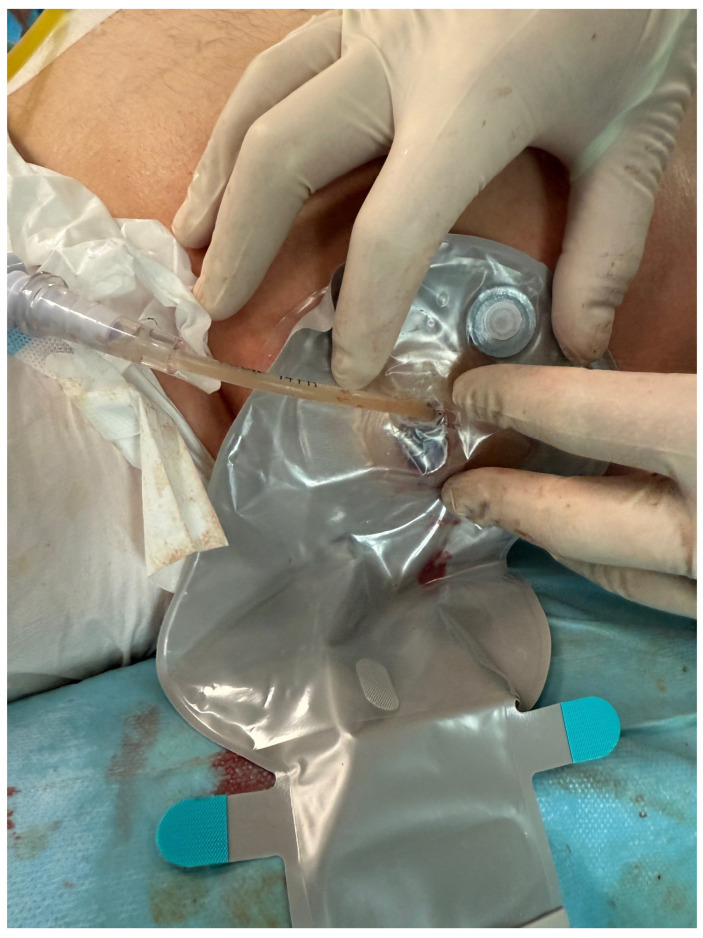
Completed percutaneous endoscopic necrosectomy, with a drainage bag applied at the site of the self-expandable stent. Source: Department of General, Gastroenterological, and Oncological Surgery, L. Rydygier Regional Hospital in Toruń.

The PEN procedure was performed under general anesthesia with endotracheal intubation in the supine position. Percutaneous endoscopic necrosectomy was performed under the guidance of ultrasound (using a Logiq P9, convex C1-6 MHz probe) and fluoroscopy. Following the establishment of percutaneous drainage, Cook Medical Acrobat 2 AWG2-35-45 guidewire was inserted and looped inside the lumen of the necrotic collection (Figure 1 and Figure 2) to be followed by implantation of a fully coated self-expandable Evolution^®^ esophageal controlled-release stent 120 mm or 150 mm in length and 20 mm in diameter (Figure 3 and Figure 4). A flexible Evis Exera III CF-H190L endoscope–gastroscope (Olympus) was then inserted into the necrotic collection through the lumen of the esophageal stent, and a percutaneous endoscopic necrosectomy procedure involving mechanical removal of necrotic tissue from the collection under direct endoscopic image guidance was performed (Figure 5, Figure 6 and Figure 7). A Dormia basket (Figure 8) was used to remove necrotic tissue. In the course of the percutaneous endoscopic necrosectomy procedure, the necrotic collection was extensively flushed with physiological saline, and the contents from the reservoir were aspirated. The PEN procedure was considered complete upon removal of demarcated necrotic tissues or upon bleeding from the inflammatory granulation tissue within the necrotic collection. If subsequent percutaneous endoscopic necrosectomy procedures were required in the same patient, the esophageal stent was left in the percutaneous position, and one or two 16 Fr silicone drains (depending on the size of the collection) were inserted into the lumen of the necrotic collection through the stent to maintain patency. The drain(s) were used to flush the collection with 100 mL of physiological saline 6 times a day. Following the completion of endoscopic treatment using percutaneous access, the esophageal stent was removed, and the stenting site was secured with a stoma bag to drain the remaining contents from the residual necrotic collection (Figure 9).

Clinical success, defined as regression of the necrotic collection and resolution of clinical symptoms associated therewith, was acknowledged in 8/9 (88.88%) patients. In 1/9 patient, surgical treatment was applied involving open-access necrosectomy following laparotomy due to the symptom of massive bleeding into the lumen of the necrotic collection. Long-term success was achieved in 7/9 (77.77%) patients. No cases of superinfection of the collections subject to percutaneous drainage or pancreatico-cutaneous fistulas were observed in the study group, whereas the cutaneous fistulas established in the course of the PEN procedure and constituting a natural consequence of the procedure closed spontaneously within 30 days after the removal of the esophageal stent.

## 3. Results

A total of 9/39 (23.07 %) patients were qualified for percutaneous endoscopic necrosectomy (PEN) via lumbar (extraperitoneal) access. The study group consisted of seven men (77.78%) and two (22.22%) women, with a mean age of 46.7 years (range 31–65 years). In 8/9 (88.89%) patients, the etiology of acute necrotizing pancreatitis and its complications was related to alcohol abuse, whereas in 1/9 (11.11%) patient, WOPN developed as a complication of cholelithiasis. In most patients, 6/9 (66.67%), the drained collections represented infected walled-off pancreatic necrosis, while in 3/9 (33.33%) cases, the WOPN collections were non-infected. In 8/9 (88.89%) patients, the necrotic collection extended predominantly along the left flank of the abdomen, and in 1/9 (11.11%) patient along the right flank. The mean largest diameter of the necrotic collections was 25.96 cm (range 18.0–35.5 cm). Active percutaneous drainage during ongoing transmural endotherapy lasted a median of 15 days (range 3-27 days). Patients underwent an average of 3.12 (range 1–7) necrosectomy sessions. A fully covered self-expandable esophageal stent with a length of 120 mm was used in 7/9 (77.8%) patients, while a 150 mm stent was implanted in 2/9 (22.2%) patients. In 6/9 (66.7%) cases, two percutaneous drains were placed, and in 3/9 (33.3%) patients, a single drain was used to maintain access and enable active lavage. Clinical success, defined as regression of the necrotic collection with resolution of related clinical symptoms, was achieved in 8/9 (88.9%) patients. Long-term success was documented in 7/9 (77.8%) patients. Among patients with documented long-term success, the mean follow-up duration was 880.7 days (range 188–1562 days). Procedure-related complications occurred in 3/9 (33.3%) patients. Bleeding associated with PEN was observed in 2/9 (22.2%) patients. In one of these cases (1/9; 11.1%), bleeding was managed conservatively with antihemorrhagic agents (etamsylate 4 × 500 mg i.v. and tranexamic acid 3 × 1000 mg i.v.) and transfusion of blood products (red blood cell concentrate and fresh frozen plasma). In the second case (1/9; 11.1%), bleeding required open surgical management (laparotomy) with necrosectomy. In one patient (1/9; 11.1%), the development of a hematoma within the left kidney and an intestinal fistula was observed; both were treated conservatively and did not require surgical intervention. No cases of superinfection of percutaneously drained collections or persistent pancreatico-cutaneous fistulas were noted; the cutaneous tracts created for PEN closed spontaneously within 30 days after esophageal stent removal (Table 1).

## 4. Discussion

Severe acute necrotizing pancreatitis is a condition that, despite the ongoing development of minimally invasive treatment techniques, is still associated with a mortality rate of up to 70% in cases of infected areas of necrosis progressing to multiple organ failure [19,20,21]. According to the currently adopted step-up strategy of management, infected and symptomatic pancreatic and peripancreatic necrotic collections require continued endoscopic, percutaneous, or surgical drainage as the ultimate solution [22].

The dual-modality drainage technique (combination of endoscopic and percutaneous drainage) involves percutaneous drainage being followed by endoscopic transmural drainage [23,24,25]. Thus, percutaneous drainage is used as the mainstay modality in the dual-modality drainage technique, with passive endoscopic percutaneous drainage being performed second in order. At the authors’ center, percutaneous drainage of pancreatic necrosis is avoided prior to endoscopic drainage. Early percutaneous drainage of pancreatic necrosis results in the evacuation of liquefied necrotic contents, which reduces the volume of the reservoir and moves the reservoir wall away from the wall of the gastrointestinal tract, which in turn precludes the possibility of percutaneous drainage [26,27]. In such cases, the patient can only be treated with percutaneous drainage, which prolongs the therapeutic process and forces earlier and more frequent necrosectomy procedures.

Notably, the choice of access to the necrotic collection should depend primarily on the location of the necrotic lesion and the experience of the medical center. In some cases, endoscopic drainage proves insufficient, or the achievement of satisfactory therapeutic results by means thereof is completely impossible. An additional access route to the necrotic collection may be necessary during endoscopic percutaneous drainage of extensive necrotic collections. This includes patients with collections extending along the left/right paracolic gutter, as well as patients with collections extending laterally and descending into the pelvis minor [28,29,30]. In this group of patients, the opportunity to access necrotic areas is provided by an innovative and minimally invasive procedure referred to as percutaneous endoscopic necrosectomy (PEN). Thanks to the use of a flexible endoscope, PEN offers the possibility of numerous endoscopic tools being used to perform necrosectomy in areas not accessible for rigid endoscopes [31].

For necrotic collections located at a greater distance from the gastrointestinal wall, PEN can be used as the next line of treatment upon the failure of percutaneous drainage from an extraperitoneal access, while providing an alternative to surgical necrosectomy from extraperitoneal access in the step-up approach. In the case of necrotic collections subject to percutaneous endoscopic drainage where the main part of the collection is located outside the omentum and penetrates into the paracolic gutters towards the pelvis, PEN, preceded by percutaneous drainage, increases the effectiveness of endotherapy of acute necrotizing pancreatitis complications [32].

A recent systematic review by Gjeorgjievski et al. [33] identified 275 percutaneous endoscopic necrosectomy cases reported in the published literature. After inclusion of the 9 cases reported in the present manuscript, the total number of published PEN now amounts to 284. At the authors’ center, the technique of percutaneous endoscopic necrosectomy was used with success in 9 patients, constituting one of the larger patient groups treated by means of this minimally invasive method. Therapeutic and long-term success was achieved in 88.8% of cases, demonstrating the efficacy and safety of PEN in this group of patients. The high efficacy of PEN in the treatment of WOPN collections extending along the pelvis and the right and left paracolic gutters, amounting to 66.67–100%, was also confirmed in two meta-analyses published in 2022 and 2023 [5,7].

The PEN technique described herein is significantly different from previously described surgical necrosectomy techniques performed using a flexible endoscope from retroperitoneal access (sinus tract endoscopy) [12,34,35]. Firstly, the PEN technique does not require mechanical widening of the percutaneous access to the collection lumen, which reduces the risk of persistent pancreatico-cutaneous fistulas. Secondly, the technique involves the use of esophageal stents, which provide a tight channel between the necrotic collection and the skin layers, reducing the risk of contents leaking from the collection into other anatomical spaces and the associated secondary infections. Thirdly, at the authors’ center, one or two 16 Fr silicone drains are inserted through the fully coated transmural endoprosthesis, facilitating additional flushing of the necrotic collection as well as the maintenance of channel patency. No plastic prostheses were placed inside the WOPN collections in any patients.

Current evidence shows that transmural endoscopic necrosectomy is considered first-line when collections are adjacent to the stomach or duodenum, offering high clinical success and lower morbidity compared to surgical approaches [35]. In cases in which access is unfavorable, video-assisted retroperitoneal debridement (VARD) and minimally invasive retroperitoneal pancreatic necrosectomy (MARPN) remain standard approaches with well-documented outcomes [36]. PEN occupies an intermediate position between purely endoscopic and surgical strategies, providing direct visualization like endoscopy but using a percutaneous route similar to VARD/MARPN. Meta-analyses report clinical success rates for PEN of 80–85% with complication rates comparable to transmural necrosectomy [33]. In our study, success achieved in 88.8% of patients aligns with these findings, suggesting that PEN may serve as a valuable alternative particularly when transmural access is not feasible.

A common technical problem with PEN consists of the difficulty of insufflating the necrotic collection during the procedure due to the large diameter of the esophageal stent relative to the diameter of the endoscope [37,38]. During the procedures performed at the authors’ center, pressing the endoscope against the lumen of the transmural prosthesis and draping the mouth of the prosthesis with a small sterile gauze pad minimized the loss of gas, thus ensuring adequate visibility throughout the endoscopic procedure. Notably, the experience of the authors’ center is based mostly, i.e., in as many as 8/9 cases (88.88%), on percutaneous endoscopic necrosectomy of collections extending along the left paracolic gutter. Drainage and necrosectomy procedures are easier and much safer when performed on the left side of the body as compared to right-sided procedures.

It should be emphasized that it is important that the surgeon performing the PEN procedure has extensive experience, especially in ultrasound-guided percutaneous drainage procedures. One of the possible complications of the PEN procedure is perirenal hematoma. Inflammatory infiltration around the WOPN collection can impair ultrasound imaging, increasing the risk of hematoma developing in the drainage area. The PEN procedure has certain limitations [39]. The flexible endoscope, as used in the procedure, may not be sufficient to remove dense necrotic tissue. In such cases, a rigid endoscope–nephroscope used as part of the MARPN procedure might be more effective. Since no relevant data is currently available in the literature, further studies should focus on direct comparisons between PEN and MARPN.

## 5. Conclusions

Percutaneous endoscopic necrosectomy (PEN) is an effective and minimally invasive method for the management of walled-off pancreatic necrosis, particularly in cases where endoscopic transmural access is not feasible or when necrotic collections extend into the paracolic gutters or toward the pelvis. Our results, in line with published data, indicate that PEN can serve as a valuable complement to existing minimally invasive approaches and may enable postponement or avoidance of surgical necrosectomy, which is associated with higher morbidity and mortality. This technique has the potential to become an important element of the step-up strategy in selected patients. However, available evidence is still based on relatively small cohorts, and further prospective multicenter studies are required to define optimal candidate selection, procedural standardization, and long-term outcomes. Despite these limitations, early clinical results remain promising and support the wider evaluation of PEN in the treatment of complications of acute necrotizing pancreatitis.

## Figures and Tables

**Table 1:** Baseline characteristics of the patient group.

No	Sex	Etiology of AP	Patient Age	Size of Necrotic Collections (cm)	Infection of WOPN	Left/Right Retroperitoneal	Size of the Esophageal Stent (mm)	Internal Plastic Stent Used	No. of PEN Session Performed	Length of Time Stent for PEN in Place	Clinical Succes and Outcomes	FU (Days)	Long-term Success
1	M	Alcoholic	33	22 × 12 cm	Infected	L	120	PTD (2)	4	8 days	Success, no complications	745	YES
2	F	Biliary	65	15 × 18 cm	Infected	L	120	PTD (2)	3	7 days	Success, bleeding	1089	YES
3	M	Alcoholic	31	22 × 32.5 cm	Infected	L	150	PTD (2)	2	3 days	Success, no complications	1562	YES
4	M	Alcoholic	54	15.2 × 19.6 cm	Infected	R	120	PTD (2)	7	26 days	Ineffective—bleeding, laparotomy	Died	NO
5	F	Alcoholic	58	19 × 22.5 cm	Non-infected	L	120	PTD (1)	1	3 days	Success, no complications	1442	YES
6	M	Alcoholic	46	28.3 × 17.44 cm	Infected	L	120	PTD (1)	3	8 days	Success, no complications	601	YES
7	M	Alcoholic	44	14.35 × 22.4 cm	Non-infected	L	120	PTD (2)	2	3 days	Success, no complications	538	YES
8	M	Alcoholic	38	25.5 × 35.5 cm	Non-infected	L	120	PTD (1)	2	7 days	Success, no complications	188	YES
9	M	Alcoholic	37	26.33 × 17.5 cm	Infected	L	150	PTD (2)	6	27 days	Success, intestinal fistula, renal hematoma		

## Data Availability

The original contributions presented in this study are included in the article/Supplementary Material. Further inquiries can be directed to the corresponding author.

## Supplementary Materials

The following supporting information can be downloaded at: https://www.mdpi.com/article/10.3390/jcm15020470/s1, CONSORT 2025 checklist item description; flowchart. Reference [40] is cited in the Supplementary Materials.

## Abbreviations

The following abbreviations are used in this manuscript:

VARD	video-assisted retroperitoneal debridement
PEN	percutaneous endoscopic necrosectomy
MARPN	minimal access retroperitoneal pancreatic necrosectomy
MIRPN	minimally invasive retroperitoneal pancreatic necrosectomy
i.v	intravenous
CT	computer tomography

## References

[1] Werge M., Novovic S., Schmidt P.N., Gluud L.L. (2016). Infection increases mortality in necrotizing pancreatitis: A systematic review and meta-analysis. Pancreatology.

[2] Copelin E., Widmer J. (2022). Management of severe acute pancreatitis in 2019. Transl. Gastroenterol. Hepatol..

[3] van Brunschot S., van Grinsven J., van Santvoort H.C., Bakker O.J., Besselink M.G., Boermeester M.A., Bollen T.L., Bosscha K., Bouwense S.A., Bruno M.J. (2018). Endoscopic or surgical step-up approach for infected necrotising pancreatitis: A multicentre randomised trial. Lancet.

[4] van Santvoort H.C., Besselink M.G., Bakker O.J., Hofker H.S., Boermeester M.A., Dejong C.H., van Goor H., Schaapherder A.F., van Eijck C.H., Bollen T.L. (2010). A step-up approach or open necrosectomy for necrotizing pancreatitis. N. Engl. J. Med..

[5] Yang Y., Zhang Y., Wen S., Cui Y. (2023). The optimal timing and intervention to reduce mortality for necrotizing pancreatitis: A systematic review and network meta-analysis. World J. Emerg. Surg..

[6] Bang J.Y., Wilcox C.M., Arnoletti J.P., Varadarajulu S. (2020). Superiority of endoscopic interventions over minimally invasive surgery for infected necrotizing pancreatitis: Meta-analysis of randomized trials. Dig. Endosc..

[7] Mohamadnejad M., Anushiravani A., Kasaeian A., Sorouri M., Djalalinia S., Houjaghan A.K., Gaidhane M., Kahaleh M. (2022). Endoscopic or surgical treatment for necrotizing pancreatitis: Comprehensive systematic review and meta-analysis. Endosc. Int. Open.

[8] Jagielski M., Jackowski M. (2021). The Role of Lumen-Apposing Metal Stents in Transmural Endoscopic Drainage of Postinflammatory Pancreatic and Peripancreatic Fluid Collections. Gastroenterol. Res. Pract..

[9] Puri R., Thandassery R.B., Alfadda A.A., Kaabi S.A. (2015). Endoscopic ultrasound guided drainage of pancreatic fluid collections: Assessment of the procedure, technical details and review of the literature. World J. Gastrointest. Endosc..

[10] Wu C.C., Martin D.T., Bauman B.D., Amateau S.K., Azeem N., Harmon J.V. (2022). Video-assisted retroperitoneal debridement for infected pancreatic necrosis: A single center series. Int. J. Surg. Case Rep..

[11] Horvath K., Freeny P., Escallon J., Heagerty P., Comstock B., Glickerman D.J., Bulger E., Sinanan M., Langdale L., Kolokythas O. (2010). Safety and efficacy of video-assisted retroperitoneal debridement for infected pancreatic collections: A multicenter, prospective, single-arm phase 2 study. Arch. Surg..

[12] Carter C.R., McKay C.J., Imrie C.W. (2000). Percutaneous necrosectomy and sinus tract endoscopy in the management of infected pancreatic necrosis: An initial experience. Ann. Surg..

[13] Goenka M.K., Goenka U., Mujoo M.Y., Tiwary I.K., Mahawar S., Rai V.K. (2018). Pancreatic Necrosectomy through Sinus Tract Endoscopy. Clin. Endosc..

[14] van Santvoort H.C., Besselink M.G., Horvath K.D., Sinanan M., Bollen T., Van Ramshorst B., Gooszen H. (2007). Dutch Acute Pancreatis Study Group. Videoscopic assisted retroperitoneal debridement in infected necrotizing pancreatitis. HPB.

[15] Szeliga J., Jackowski M. (2014). Minimally invasive procedures in severe acute pancreatitis treatment-assessment of benefits and possibilities of use. Wideochir. Inne Tech. Maloinwazyjne..

[16] Bang J.Y., Lakhtakia S., Thakkar S., Buxbaum J.L., Waxman I., Sutton B., Memon S.F., Singh S., Basha J., Singh A. (2024). Upfront endoscopic necrosectomy or step-up endoscopic approach for infected necrotising pancreatitis (DESTIN): A single-blinded, multicentre, randomised trial. Lancet Gastroenterol. Hepatol..

[17] Valentin C., Le Cosquer G., Tuyeras G., Culetto A., Barange K., Hervieu P.-E., Carrère N., Muscari F., Mokrane F., Otal P. (2024). Step-up approach for the treatment of infected necrotising pancreatitis: Real life data from a single-centre experience with long-term follow-up. BMC Gastroenterol..

[18] Jagielski M., Chwarścianek A., Piątkowski J., Jackowski M. (2022). Percutaneous Endoscopic Necrosectomy-A Review of the Literature. J. Clin. Med..

[19] Leonard-Murali S., Lezotte J., Kalu R., Blyden D.J., Patton J.H., Johnson J.L., Gupta A.H. (2021). Necrotizing pancreatitis: A review for the acute care surgeon. Am. J. Surg..

[20] Jain S., Mahapatra S.J., Gupta S., Shalimar, Garg P.K. (2018). Infected Pancreatic Necrosis due to Multidrug-Resistant Organisms and Persistent Organ failure Predict Mortality in Acute Pancreatitis. Clin. Transl. Gastroenterol..

[21] Büchler M.W., Gloor B., Müller C.A., Friess H., Seiler C.A., Uhl W. (2000). Acute necrotizing pancreatitis: Treatment strategy according to the status of infection. Ann. Surg..

[22] Mukai S., Sofuni A., Tsuchiya T., Tanaka R., Tonozuka R., Matsunami Y., Nagai K., Kojima H., Minami H., Hirakawa N. (2025). Endoscopic Step-up Approach for Walled-off Necrosis After Acute Pancreatitis. DEN Open.

[23] Gluck M., Ross A., Irani S., Lin O., Gan S.I., Fotoohi M., Hauptmann E., Crane R., Siegal J., Robinson D.H. (2012). Dual modality drainage for symptomatic walled-off pancreatic necrosis reduces length of hospitalization, radiological procedures, and number of endoscopies compared to standard percutaneous drainage. J. Gastrointest. Surg..

[24] Ross A.S., Irani S., Gan S.I., Rocha F., Siegal J., Fotoohi M., Hauptmann E., Robinson D., Crane R., Kozarek R. (2014). Dual-modality drainage of infected and symptomatic walled-off pancreatic necrosis: Long-term clinical outcomes. Gastrointest. Endosc..

[25] Bomman S., Sanders D., Coy D., La Selva D., Pham Q., Zehr T., Law J., Larsen M., Irani S., Kozarek R.A. (2023). Safety and clinical outcomes of early dual modality drainage (<28 days) compared to later drainage of pancreatic necrotic fluid collections: A propensity score-matched study. Surg. Endosc..

[26] Jagielski M., Piątkowski J., Jackowski M. (2022). Early endoscopic treatment of symptomatic pancreatic necrotic collections. Sci. Rep..

[27] Ramesh S., Verma Y., Perera Molligoda Arachchige A.S. (2023). Early vs. late percutaneous catheter drainage of acute necrotic collections in patients with necrotizing pancreatitis. Abdom. Radiol..

[28] Saumoy M., Kumta N.A., Tyberg A., Brown E., Lieberman M.D., Eachempati S.R., Winokur R.S., Gaidhane M., Sharaiha R.Z., Kahaleh M. (2018). Transcutaneous Endoscopic Necrosectomy for Walled-off Pancreatic Necrosis in the Paracolic Gutter. J. Clin. Gastroenterol..

[29] Vyawahare M.A., Gulghane S., Titarmare R., Bawankar T., Mudaliar P., Naikwade R., Timane J.M. (2022). Percutaneous direct endoscopic pancreatic necrosectomy. World J. Gastrointest. Surg..

[30] Binda C., Perini B., Coluccio C., Giuffrida P., Fabbri S., Gibiino G., Vizzuso A., Giampalma E., Fabbri C. (2024). Metal stent and percutaneous endoscopic necrosectomy as dual approach for the management of complex walled-off pancreatic necrosis. Minerva Surg..

[31] Jürgensen C., Brückner S., Reichel S., Kilian M., Pannach S., Distler M., Weitz J., Neser F., Hampe J., Will U. (2017). Flexible percutaneous endoscopic retroperitoneal necrosectomy as rescue therapy for pancreatic necroses beyond the reach of endoscopic ultrasonography: A case series. Dig. Endosc..

[32] Binda C., Sbrancia M., La Marca M., Colussi D., Vizzuso A., Tomasoni M., Agnoletti V., Giampalma E., Ansaloni L., Fabbri C. (2021). EUS-guided drainage using lumen apposing metal stent and percutaneous endoscopic necrosectomy as dual approach for the management of complex walled-off necrosis: A case report and a review of the literature. World J. Emerg. Surg..

[33] Gjeorgjievski M., Bhurwal A., Chouthai A.A., Abdelqader A., Gaidhane M., Shahid H., Tyberg A., Sarkar A., Kahaleh M. (2023). Percutaneous endoscopic necrosectomy (PEN) for treatment of necrotizing pancreatitis: A systematic review and meta-analysis. Endosc. Int. Open.

[34] Trikudanathan G., Hashmi H., Dirweesh A., Amateau S., Azeem N., Mallery S., Freeman M.L. (2020). Rendezvous transgastric and percutaneous sinus tract endoscopy (STE) for debridement of necrotic collections with deep retroperitoneal extension: A case series. Endosc. Int. Open.

[35] Feng L., Guo J., Wang S., Liu X., Ge N., Wang G., Sun S. (2021). Endoscopic transmural drainage and necrosectomy in acute necrotizing pancreatitis: A review. J. Transl. Intern. Med..

[36] Liu Z.W., Yang S.Z., Wang P.F., Feng J., He L., Du J., Xiao Y., Jiao H., Zhou F., Song Q. (2020). Minimal-access retroperitoneal pancreatic necrosectomy for infected necrotizing pancreatitis: A multicentre study of a step-up approach. Br. J. Surg..

[37] Li D.L., Zhang H., Lv J., Quan L., Liu D., Zhao L., Liu B. (2024). Percutaneous endoscopic necrosectomy with the assistance of implanted stent to manage walled-off necrosis: First clinical experience. Endoscopy.

[38] Oshiro K., Hara K., Ogata T., Koda H., Okuno N., Haba S., Kuwahara T. (2025). Percutaneous endoscopic necrosectomy for extensive walled-off necrosis using two endoscopes simultaneously: A report of two cases. Clin. J. Gastroenterol..

[39] Zheng X., Li L., Li J., Huang X., Le Y., Ke H., Wu Y., Shu X., Liu Z., Xia L. (2021). Risk factors for bleeding in patients with acute necrotizing pancreatitis undergoing endoscopic necrosectomy. HPB.

[40] Hopewell S., Chan A.W., Collins G.S., Hróbjartsson A., Moher D., Schulz K.F., Tunn R., Aggarwal R., Berkwits M., Berlin J.A. (2025). CONSORT 2025 statement: Updated guideline for reporting randomised trials. BMJ.

