# Supplementary figures and images for: Percutaneous Endoscopic Necrosectomy of Walled-Off Pancreatic and Peripancreatic Necrosis
Source: J Clin Med. 2026 Jan 7;15(2):470. doi: 10.3390/jcm15020470 (PMC12842036; doi:10.3390/jcm15020470)

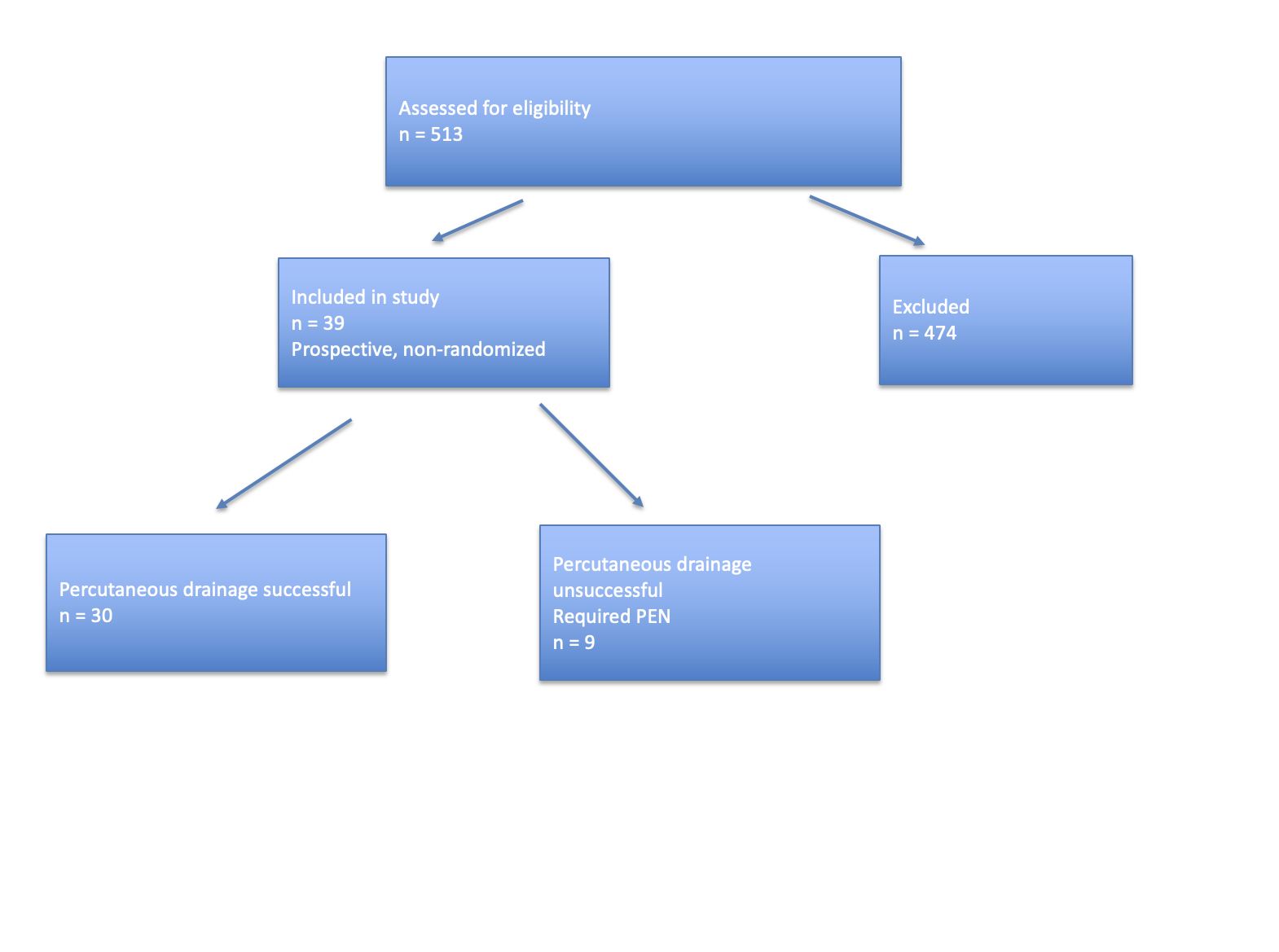

Supplement: Supplementary file 1 [file jcm-15-00470-s001.zip › jcm-4028200-flowchart.png]
